# Adaptive filtering of microarray gene expression data based on Gaussian mixture decomposition

**DOI:** 10.1186/1471-2105-14-101

**Published:** 2013-03-20

**Authors:** Michal Marczyk, Roman Jaksik, Andrzej Polanski, Joanna Polanska

**Affiliations:** 1 Institute of Automatic Control, Silesian University of Technology, Gliwice 44-100, Poland; 2 Institute of Informatics, Silesian University of Technology, Gliwice 44-100, Poland

## Abstract

**Background:**

DNA microarrays are used for discovery of genes expressed differentially between various biological conditions. In microarray experiments the number of analyzed samples is often much lower than the number of genes (probe sets) which leads to many false discoveries. Multiple testing correction methods control the number of false discoveries but decrease the sensitivity of discovering differentially expressed genes. Concerning this problem, filtering methods for improving the power of detection of differentially expressed genes were proposed in earlier papers. These techniques are two-step procedures, where in the first step some pool of non-informative genes is removed and in the second step only the pool of the retained genes is used for searching for differentially expressed genes.

**Results:**

A very important parameter to choose is the proportion between the sizes of the pools of removed and retained genes. A new method, which we propose, allow to determine close to optimal threshold values for sample means and sample variances for gene filtering. The method is adaptive and based on the decomposition of the histogram of gene expression means or variances into mixture of Gaussian components.

**Conclusions:**

By performing analyses of several publicly available datasets and simulated datasets we demonstrate that our adaptive method increases sensitivity of finding differentially expressed genes compared to previous methods of filtering microarray data based on using fixed threshold values.

## Background

In DNA microarray experiments the expression pattern of many thousands of genes is discovered, which gives the possibility to reflect biological states of cells. The primary use of DNA microarrays is the identification of genes expressed differentially between various conditions. Differentially expressed genes (DEGs) can be identified on the basis of different criteria; most often they are identified on the basis of p-values of statistical tests. DEGs are important characteristics of experimental results; they are summed up in the so called gene signatures and are further used in many contexts. The efficiency of identification of DEGs can be further verified e.g., by conducting sample classification experiments based on gene expression signatures selected from the top differentially expressed genes. The problem of construction of gene expression signatures for their use in molecular classifiers was studied in numerous papers; the discussion of many useful ideas can be found in [1].

A challenge in identification of DEGs based on DNA microarray data is a dimensionality problem; a small number of samples versus tens of thousands of genes’ expressions measured in each sample. A large number of statistical tests for finding DEGs result in the occurrence of many false discoveries among genes called differentially expressed. This problem can further manifest itself in the irreproducibility of results of different studies, e.g., a DEG discovered in one study is not found in another one, or a molecular classifier designed in one study does not properly predict sample status for analogous data collected in another study. The proportion of false discoveries among genes called differentially expressed (false discovery rate, FDR) can be controlled by using corrections for multiple testing [2,3]. However, introducing limits on FDR leads to the decrease of sensitivity of the procedure for discovering DEGs.

Concerning the above described problem, methods for increasing the detection power for DNA microarray data, i.e., for improving sensitivity of the process of the discovery of DEGs while keeping the FDR under control, have been proposed in earlier papers [4-10]. These methods are based on two-step procedures, where the first step is pre-selection (filtering) aimed at removing some pool of non-informative genes, and the second step is the discovery of DEGs in the pool of retained genes. If the pool of genes removed in the first step includes no or only few DEGs, the detection power of the process of discovery of DEGs becomes improved.

Methods for increasing the detection power for DEGs, proposed in earlier papers, can be grouped according to criteria used for filtering out non-informative genes. The first group includes methods based on introducing thresholds for means or variances of gene expression signals. This approach was studied in several papers [4-10], and it has been shown that for certain ranges of threshold values for means or variances of gene expressions (or their base two logarithms) filtering increases sensitivity of discovery of DEGs. The second group includes methods based on detection calls (labels) assigned to probe sets by the Affymetrix MAS 5.0 signal pre-processing procedure. These labels are aimed at indicating whether the specific mRNA is detectable (perfect matches show a higher hybridization signal than their corresponding mismatches) based on rejecting the null hypothesis in the Wilcoxon signed rank test. A method based on probe detection calls, proposed in [4], removes all genes except the fraction called ‘Present’ in at least one group of samples. Hackstadt and Hess [7] compared detection call methods and methods based on overall mean and variance filtering (in log2 and original scale) on a probe set level in different combinations with the two FDR control methods and three pre-processing methods. They have discovered that both filtering methods, by detection call and variance (on the original scale) paired with either of the false discovery rate multiple testing correction methods considered led to an increase in the number of differentially expressed genes identified. The third group includes methods based on fitting statistical models to probe sets expression data [5,6,10]. These models can be factor analysis models [6] or principal component analysis (PCA) models [10], which explore sources (components) of variation in the data and allow the researchers to retain only the genes corresponding to components with large enough variation. A method named “I/NI - calls”, proposed by Talloen et al. [6], is based on approximating the probe intensity values by products of unknown loadings and factors. The authors of [6] assume normal priors for loadings and estimate probe set signal variation by the variance of the hidden factor (given data). They call a probe set informative if the variance of the hidden factor exceeds the assumed threshold. Lu et al. [10] propose another, simpler strategy to filter out non-informative genes (probe sets), which uses PCA analysis on the probe-level data. They call their method PVAC (proportion of variation accounted by the first principal component) and use variability captured by the first principal component as a measure of consistency among probes in a probe set and consequently as a threshold for filtering out genes. The PVAC method shows sensitivity comparable to the method reported by Talloen et al. [6] but its use offers several advantages. It does not rely on any distribution assumptions, no selection of informative prior is required. The approach is also computationally simpler and therefore more practical.

Two-step procedures for DEGs discovery should be constructed in such a way that the first step of gene filtering is nonspecific (blind on class labels), i.e., information on the samples’ class labels is ignored. Otherwise the control of FDR becomes lost. In a recent paper by Bourgon et al. [8], they derive a more restrictive and precise “marginal independence” condition, which states that the criterion for gene filtering in the first step and test statistics for DEGs discovery in the second step should be independent under the null hypothesis. A violation of this condition can again lead to the loss of FDR control. A group of two-step procedures for DEGs discovery constructed in such a way that the first step is based on setting thresholds on sample means or variances and the test statistics in the second step is given by the t distribution was proven in [8] to satisfy the marginal independence condition. Therefore these methods are of special interest, due to the reliability of the estimated values of sensitivities and FDRs.

A basic parameter in these methods is the size of the pool of genes to be filtered out. The choice of this parameter is of crucial importance since filtering out too few genes does not improve the sensitivity enough, while filtering out too many genes can lead to the removal of DEGs together with non-informative genes. Papers [7] and [10] address the problem of the choice of the size of the pool of genes to be filtered out. In [7] two methods for specifying this number are considered. In the first method, the number of genes to be filtered out is estimated on the basis of the number of probe sets labeled “Absent” by the Affymetrix MAS 5.0 signal pre-processing procedure. Authors of [7] also recommend another, simpler method of filtering out 50% of probe sets. Lu et al. [10] also use this recommendation for filtering by overall mean or variance. However, different datasets may contain different numbers of non-informative genes, so using a fixed proportion (50% or some other value) of filtered out genes may lead to the loss of efficiency of the filtration method. Therefore, in the paper we address the problem of adaptive choice of the size of the pool of genes to be filtered out. By adaptive choice we mean the approach with the threshold level for filtering depending on the probability distribution of the analyzed signal (sample mean or variance). We propose a method based on the decomposition of the probability density function into a mixture of Gaussian components and on the hypothesis that the gene content of the Gaussian components is meaningful with respect to informative versus non-informative status of genes. We use the maximum likelihood method with the EM algorithm to obtain decompositions computationally. We also compare results of our adaptive filtering method to results obtained in references [7] and [10].

## Methods

Analogously to [7] and [10] we consider gene filters based on sample mean and sample variance in either log2 or original scales. We use the following abbreviations for naming different filtering methods: NF – no filter, S – signal mean-intensity-based filter, V – variance-based filter, LV – variance-based filter calculated on the log2 scale data. The letter “A” as a prefix corresponds to adaptive version of the filter, underscore with a given number P as a suffix corresponds to the fixed percentage P of genes filtered out. For example, ALV represents (our) adaptive method used for variance calculated on the log2 scale expressions, while V_50 represents the method of filtering out 50% genes based on setting a threshold for variance calculated on the original scale.

### Data

For testing performance of different filtering methods, we use four datasets previously analyzed in the referenced papers: an artificially created dataset, a spike-in dataset, a rat diabetes dataset and a leukemia dataset.

The artificially created dataset is produced by using the same method as that described in [7]. All distribution parameters are set to the same values. Two scenarios for simulations are (i) expression signals independent between genes and (ii) the signal values between genes follow a “clumpy dependence” [7,11]. The simulated data include two groups of five samples with signal values generated for 50,000 genes for each sample. The number of true equally expressed genes (EEGs) varies from 70% to 95%. In both scenarios, the simulation is repeated 50 times.

The spike-in dataset (Gene Expression Omnibus (GEO) database accession number GSE21344) consists of 18 Affymetrix Drosophila Genome 2.0 microarrays (with 18,952 probe sets) representing two different conditions, each of which contains 5,749 identical cRNAs at different relative concentrations. For each condition, the total amount of cRNA is the same, and there are similar numbers of up- and down-regulated cRNAs: 1,146 individual RNAs are up- and 947 are down-regulated, with known fold changes varying between 1.2 and 4, and 3,643 RNAs are identical in abundance between the two conditions. The amount of RNA hybridized to the arrays in the current experiment is calibrated such that the gene intensities fell within the range commonly seen in experiments stored in GEO [12].

The rat diabetes dataset (GEO accession number GSE5606) was obtained in an experiment conducted to detail global changes that occur in gene expression in the left ventricular of rat hearts related to streptozotocin-induced diabetes [13]. Expression profiles were recorded sixteen weeks after induction. Samples obtained from seven animals from each of the groups (case and control) were hybridized to an Affymetrix Rat Genome 230 2.0 GeneChip (with 31,099 probe sets).

The leukemia dataset comes from a microarray experiment on the Affymetrix HG-U95Av2 platform (12,625 probe sets) done on the pretreatment leukemia samples from bone marrow and/or peripheral blood. Molecular diagnostic studies confirmed the presence of BCR/ABL gene rearrangements in 37 patients. Forty six cases did not harbor any major molecular abnormality [14].

All datasets used in this study were previously published and are publicly available either in the GEO database or on the author’s web site. Researches involving human participants [14] and animals [13] fulfilled requirements concerning informed consents of participants and ethical approval by appropriate institutions.

### Microarray normalization procedure

Microarray normalization is done by using the robust multichip average algorithm RMA [15] that includes background correction, quantile normalization and summarization by the median polish approach. The RMA procedure includes log2 transformation. If necessary, in order to obtain the original scale we perform the inverse transformation – the base 2 power function.

### Gaussian mixture decomposition

The analyzed signal, denoted by *x*, assigned to each probe set of the microarray chip corresponds to the mean or variance of gene expressions computed over the samples. In the case when *x* corresponds to the sample mean (S - method) the expression signal is log2 transformed. In the case when *x* corresponds to the sample variance, two further possibilities are considered, (i) the expression signal is log2 transformed and then *x* is computed as the logarithm of the sample variance (LV - method), (ii) the original expression signal is used and then *x* is computed as the logarithm of the sample variance (V - method). The logarithm transformation is aimed at reducing skewness of distributions of sample variances. Genes/probe sets on the microarray chip are numbered 1, 2 … *N*. *N* is the total number of genes/probe set on the microarray chip. The value of the signal *x* corresponding to gene/probe set no. *n* is denoted by *x*_*n*_.

Let *f(x)* denote the probability density function corresponding to the analyzed signal *x*. The Gaussian mixture decomposition model (GMM) of *f(x)* is:

$$f\left(x\right)=\sum_{k=1}^{K}\alpha_{k}f_{k}\left(x,\mu_{k},\sigma_{k}\right),\;\sum_{k=1}^{K}\alpha_{k}=1.$$

In the above expression *K* stands for the number of Gaussian components, *α*_*k*_ are non-negative component weights, *f*_*k*_ is the probability density function of the *k-th* Gaussian component:

$$f_{k}\left(x,\mu_{k},\sigma_{k}\right)=\frac{1}{\sigma_{k}\sqrt{2\pi }}\exp\left[-\frac{{\left(x-\mu_{k}\right)}^{2}}{2\sigma_{k}^{2}}\right]$$

and *μ*_*k*_, *σ*_*k*_ are *k-th* Gaussian component parameters – mean and standard deviation.

The Gaussian mixture model is fitted to the experimental expression mean intensity or variance by using the method of maximization of the log-likelihood function:

$$\log L=\sum_{n=1}^{N}\ln\sum_{k=1}^{K}\alpha_{k}f_{k}\left(x_{n},\mu_{k},\sigma_{k}\right)$$

The expectation maximization (EM) algorithm [16] for recursive maximization of the likelihood function is applied. The initial values for decomposition parameters are randomly generated.

For the cases of analyses of the spike-in dataset, the rat diabetes dataset and the leukemia dataset we estimate the number of components *K* in the mixture distribution by launching EM recursions many times with different *K*, and using the Bayesian information criterion (BIC) [17] evaluated using values of parameters found in EM procedure:

$$\mathrm{BIC}=-2\;*\;\log L+\left(3\;*\;K-1\right)\;*\log\left(N\right)$$

The estimated value of the number of components *K* corresponds to the smallest value of BIC, searched over the range from 1 to 15. For large *N*, the BIC criterion leads to quite reliable estimates of the numbers of components [17]. After the decomposition of the probability density function, each gene is assigned to one of the Gaussian components by using the maximum a posteriori (MAP) rule [16]. In other words, if *x*_*n*_ is the signal value corresponding to the gene *n*, then this gene is assigned to component number *k* if *α*_*χ*_*f*_*χ*_(*x*_*n*_, *μ*_*χ*_, *σ*_*χ*_) takes the maximal value for *χ* = *k*.

In the case of simulated data we take two approaches. In the first approach we assume that estimating the number of Gaussian components by using the BIC criterion is not necessary since the scenario of the simulation experiment imposes existence of two groups of genes. We therefore decompose the distribution of the signal *x* into a fixed number of 2 Gaussian components. In the second approach we use the same method of estimating number of components *K* as the one described in the previous paragraph. It should be noted that, in the second approach, in each of multiple repetitions of simulation experiment, the estimated number of components *K* can be different.

### Gene filtering

Our method for gene filtering involves removing genes belonging to components, which we expect to contain mostly non-informative genes. It is known that genes corresponding to either low values of mean expression or to low values of variance of expression are more likely to be non-informative [4-10]. The same property should pertain to Gaussian components. When we decompose the sample means or sample variances into Gaussian components, we can order the components with respect to their location parameter (mean of the Gaussian component). Then we remove genes which belong to components located at the left hand side of the signal scale, i.e., with the lowest values of this parameter. We assume that their inclusion into the further analysis would lead rather to false discoveries than to detection of true DEGs.

The problem is how many components corresponding to low values of x should be removed. We propose and analyze two methods for choosing the number of components to remove. The first one is based on the “top three” rule (in the further text denoted by using abbreviation “top3”). More specifically, we assume that three components with highest values for parameter of location, called high-level expressed genes, medium-level expressed genes and low-level expressed genes, are informative and we retain genes corresponding to these components. Other genes are removed. The second method is to use a clustering procedure, which classifies estimated Gaussian components into two groups. We have chosen k-means clustering in three dimensional space with coordinates given by means, standard deviations and weights of Gaussian components. The K-means algorithm minimizes the within-cluster sums of squared Euclidean distances from each point to the center of the cluster. The number of clusters is assumed equal to 2. Two three dimensional clusters are ordered with respect to their location along the “mean of Gaussian component” coordinate. Then the cluster which location along this coordinate corresponds to a smaller value is considered non-informative. Consequently, genes that belong to the Gaussian components within this cluster are removed.

In certain situations we have to use adjustments of the filtering methods described above depending on K. Namely, if the number of components is *K = 2* or *K = 3*, which can result from estimation, then the top3 method is considered as equivalent to NF. The clustering method works properly in all situations where *K ≥ 2*. In all computations we have never encountered the situation where *K = 1*. However, if encountered, both methods top3 and k-means would be equivalent to NF.

### Discovery of DEGs, correction for multiple testing

For discovery of DEGs we use the two-sample *t*-test with equal variances, as in other studies [7,10]. For multiple testing correction we use the procedure for controlling FDR introduced by Storey and Tibshirani, called further q-value FDR correction [3]. The FDR constraint equal to 0.05 is used.

### Assessment of the detection power of methods for discovery of DEGs

For the artificially created dataset and the spike-in dataset true differential expression status of each probe set is known, so in experiments with a various t-statistics threshold for these data sets we can always figure out which of the detected DEGs are true and which are false. We illustrate and compare detection powers achieved by investigated methods by using the following indexes: receiver operating characteristic curve (ROC) [18] plotted in the coordinate system sensitivity versus FDR, area under the ROC curve (AUC) and the F1 measure defined as the harmonic mean of 1-FDR and sensitivity.

$$F1=\frac{2\;*\;\left(1-\mathrm{FDR}\right)\;*\;\mathrm{Sensitivity}}{1-\mathrm{FDR}+\mathrm{Sensitivity}}$$

Larger values of F1 measure suggest better performance of the method. It takes the maximum value 1 for sensitivity equal to 1 and FDR equal to 0.

For the artificially created dataset, we additionally change the structure of the simulated data by assuming different proportions between EEGs and DEGs, and we study their influence on the detection power of different methods. For experimental datasets, where the true differential expression status of probe sets is not known, for comparing different filtering methods we use the index proposed in [8], defined by the number of null hypotheses which can be rejected in the set of genes remaining after filtering under a given constraint on FDR.

### Computational environment, developed scripts

All calculations and analyses were done in MATLAB 7.11 environment by MathWorks. All script files are available on request from the authors (Joanna.Polanska@polsl.pl).

## Results

In this section, we present the results of using decompositions of distributions of sample means and variances into Gaussian components for gene filtering. We also compare these results to results obtained with gene filtering methods reported in [7] and [10].

### Gaussian mixture decompositions of sample means and variances

**Figure 1:**
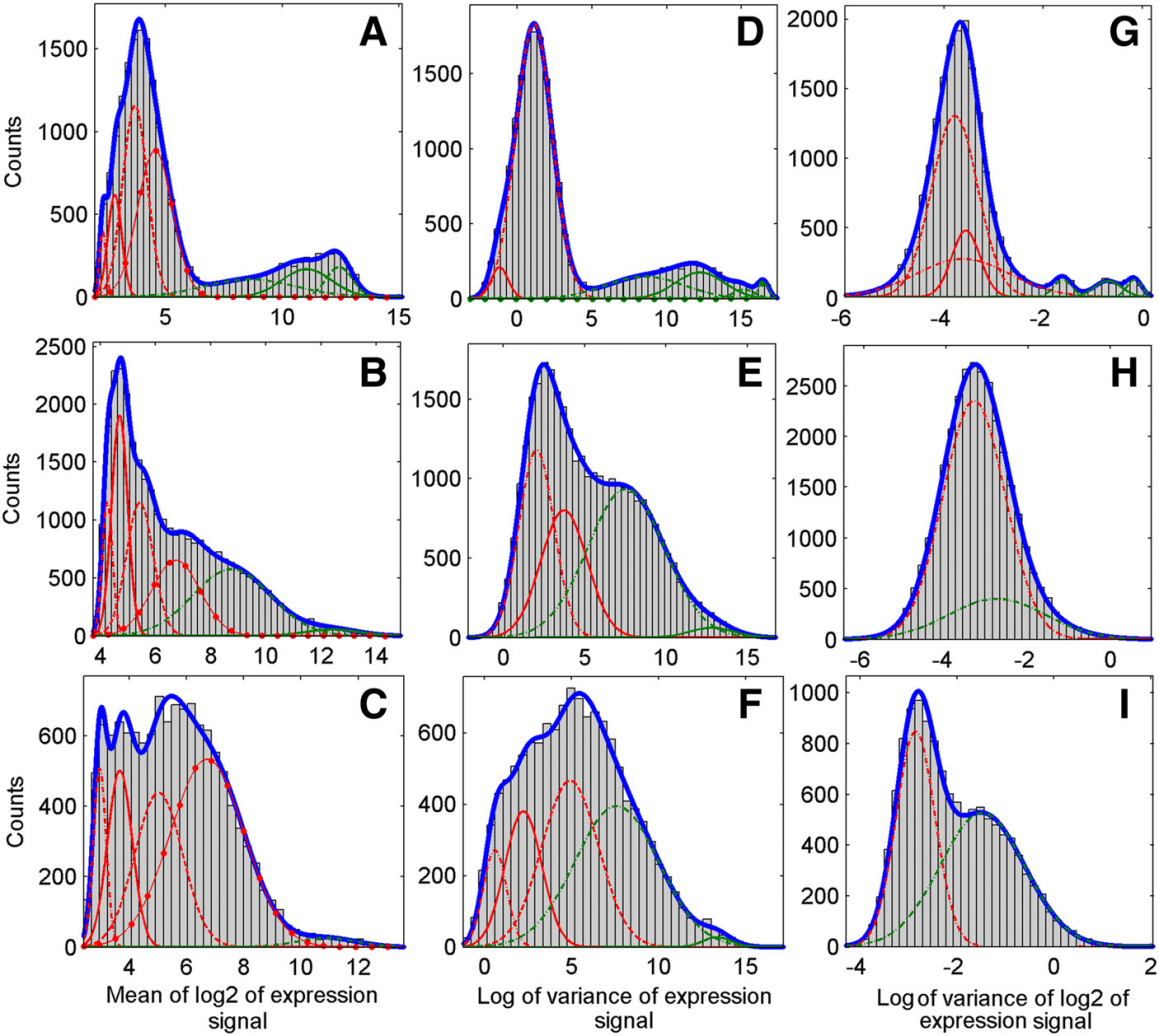
**Histograms of gene expression means and variances for analyzed datasets and their Gaussian mixture models.** Rows correspond to datasets, first row (**A**, **D**, **G**) – spike in dataset, second row (**B**, **E**, **H**) – rat diabetes dataset, third row (**C**, **F**, **I**) – leukemia dataset. Columns correspond to signals, first column – sample mean signal (S – method), second column – sample variance in the original scale signal (V – method), third column – sample variance in the log2 scale (LV –method). Probability density functions corresponding to separate Gaussian components are drawn with the use of different line styles, red color corresponds to components assigned to removal and green color – to components assigned to retain. Removal or retaining is decided by using the k-means method (explanations in the text). Plots of probability density function of mixture models given by sums of probability density functions of components are drawn in blue.

In Figure 1, we present histograms of sample means and variances of the expression data and their Gaussian mixture decompositions obtained by using EM iterations and the BIC criterion. The numbers of Gaussian components obtained in each of these computational experiments are reported as entries of Table 1, where in parentheses, we also give the numbers of Gaussian components corresponding to high levels of signal x, retained as informative using the k-means method. The rationale for declaring Gaussian components informative or non-informative is described in the Methods section. For simulated data we did not apply the LV method because the scenario of their generation already implies the expression values range corresponding to the logarithmic scale. Computing the logarithm (again) creates data unsuitable for rational interpretation. In [7] the same approach was used.

**Table 1:** Numbers of Gaussian components obtained using the BIC criterion

**Dataset**	**S**	**V**	**LV**
Spike-in	7 (3)	6 (4)	6 (3)
Diabetes	6 (2)	4 (2)	2 (1)
Leukemia	5 (1)	5 (2)	2 (1)

Entries in the table present the number of Gaussian components in the estimated mixture distributions. Additionally, in parentheses, the number of Gaussian components retained as informative based on the k-means method, are given.

### Comparisons of detection powers of algorithms with different filtration methods

**Figure 2:**
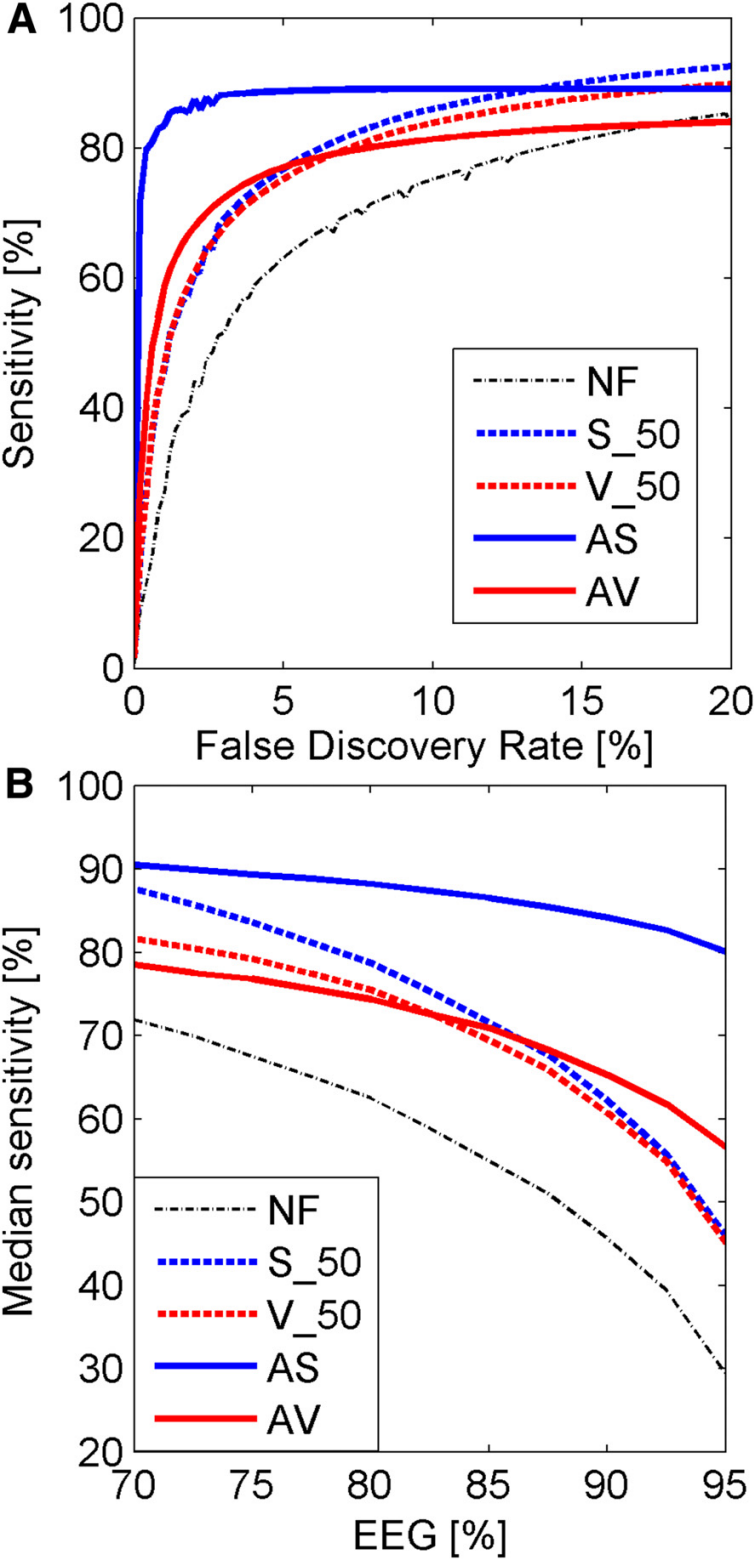
**Comparison of different filtering methods for simulated data.** Upper panel (**A**): ROC curves (computed by averaging over the 50 simulations) corresponding to different filtering methods in the simulated dataset. The proportion between informative and non-informative genes was set to 85% EEGs versus 15% DEGs. Lower panel (**B**): Change of median sensitivity at 5% FDR calculated across 50 iterations, resulting from the change of proportions between EEGs and DEGs from 70% to 95% in the simulated dataset. Different colors correspond to different filtering types of filters: red color is assigned to the filtration in sample variance domain, blue – sample mean, and black – no filtration. Different line styles correspond to different methods: solid line shows the results for adaptive filtering, dashed line – fixed threshold filtering.

The artificially created dataset contains 10 artificial samples (5 cases and 5 controls) created by using the simulation algorithm described in [7]. For two scenarios considered, the scenario of expressions simulated independently for each gene and the scenario of the “clumpy dependence” between expressions of different genes, the results of analyses and comparisons are rather similar. One difference is that dispersions of both sensitivities and FDRs obtained across 50 simulations are higher in the dataset with “clumpy dependence”. Therefore, we report only results concerning the “clumpy dependence” simulation model. In Figure 2 we present comparisons of efficiencies of detection of DEGs in the simulated dataset for two approaches, fixed 50% filtering threshold on sample means and variances proposed in [7] and our adaptive approach based on Gaussian mixture decompositions into a fixed number of 2 Gaussian components. In the upper panel of Figure 2, we show ROC curves (FDR versus sensitivity, computed by averaging over the 50 simulations) corresponding to different filtration methods (S_50, V_50, AS, AV), obtained for the case where the proportion between informative and non-informative genes was set to 85% EEGs versus 15% DEGs. We also plot a ROC curve corresponding to the case where no filtering step is applied (NF). We have also studied the influence of the proportion between DEGs and EEGs in the dataset on the median sensitivity (computed over 50 simulations) achieved by different filtering methods at 5% FDR. We present median sensitivities versus the percent of EEGs in the lower panel (B) of Figure 2. Results of applying adaptive filtration based on the Gaussian decomposition method with the estimated number of components are reported in Table 2.

**Table 2:** Comparison of results of using different filtering methods applied to the artificially created dataset

**Method**	**NF**	**S_50**	**V_50**	**Fixed**		**Top3**		**K-means**	
				**AS**	**AV**	**AS**	**AV**	**AS**	**AV**
AUC	12.47	15.14	14.59	17.14	14.10	12.47	14.08	**17.41**	14.07
Sensitivity	54.73	71.48	69.33	86.54	70.35	54.74	66.08	**87.81**	66.02

Results illustrated by the area under the ROC curve (average AUC over 50 simulations) from FDR equal 0 to 20% and median sensitivity at 5% FDR calculated across 50 simulations for the case when the proportion between informative and non-informative genes was set to 85% EEGs versus 15% DEGs.

**Figure 3:**
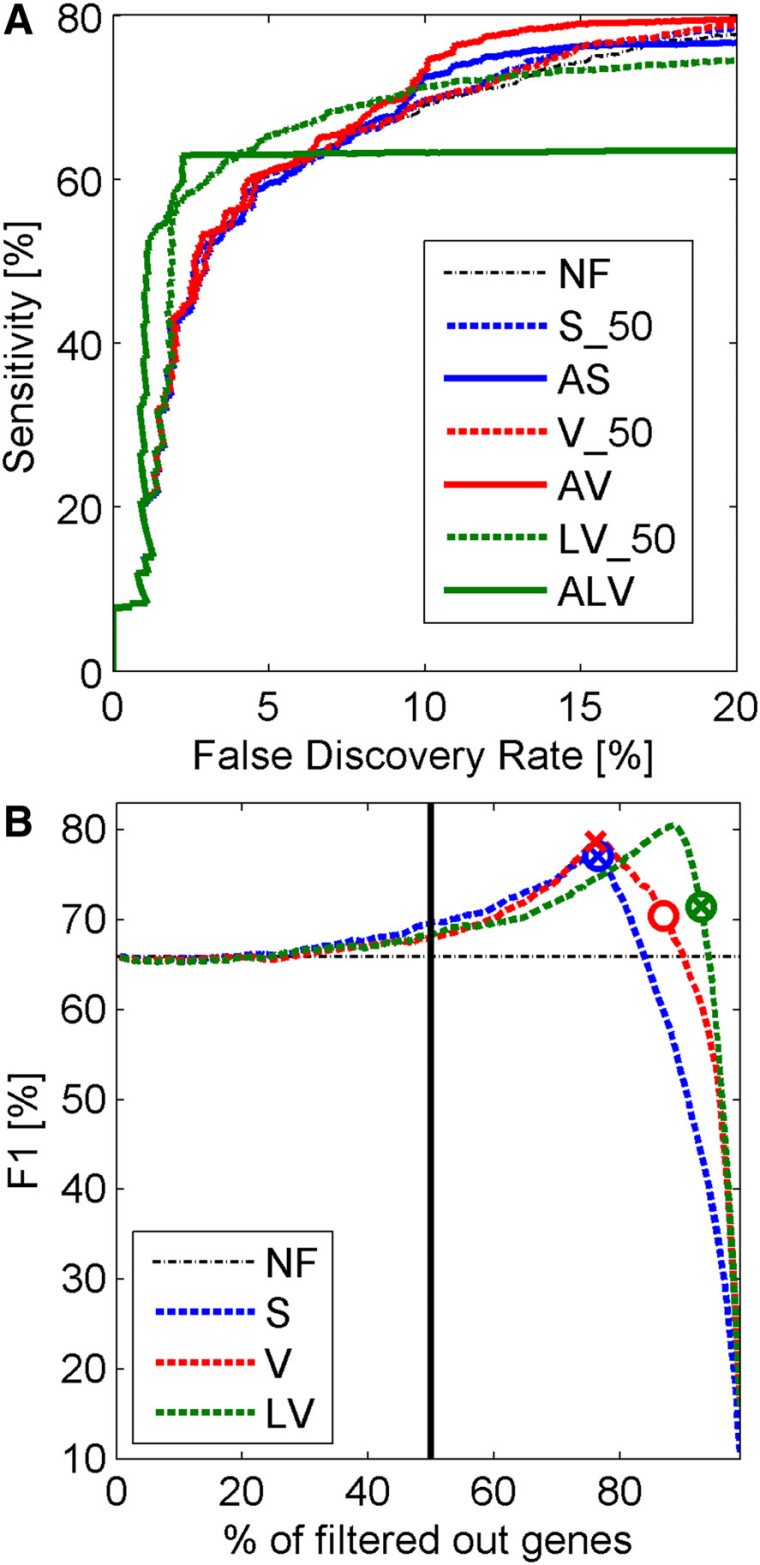
**Comparisons of different filtering methods for spike-in data.** Upper panel (**A**): ROC curves for different filtering methods. Adaptive filtering results are based on k-means method. The line representing S_50 filtering method, in the upper panel (**A**) is hard to notice due to the fact that it is obstructed by other lines. Lower panel (**B**): Change of F1 measure versus percentage of genes filtered out by different filtering methods. 50% threshold is additionally marked with vertical black line. Circles and x-signs on the plot correspond to points given by percentages following from our adaptive filtering methods (top3 and k-means respectively) and corresponding values of the F1 measure. Different colors correspond to different filtering types of filters: red color is assigned to the filtration in sample variance domain, blue – sample mean, green – sample variance in log scale, and black – no filtration. Different line styles correspond to different methods: solid line shows the results for adaptive filtering, dashed line – fixed threshold filtering. Percentages of the removed genes after AS, AV and ALV filtering are 76.4, 86.9 and 92.8, respectively, for the top3 method, and 76.4, 76.1 and 92.8, respectively, for the k-means method.

In the first step of the analysis of the spike-in dataset, we compare the results of gene filtering methods with the percentage of genes to filter out recommended in [7] (S_50, V_50, LV_50) to our adaptive methods based on GMM (AS, AV, ALV). As a reference, we also show the results of DEGs discovery for the case where no filtering step is applied (NF). Comparisons between different methods concern ROC curves (FDR versus sensitivity), which are shown in the upper plot (A) of Figure 3 and F1 measures shown in the lower plot (B) of Figure 3. The adaptive filtration methods AS, AV, ALV used for producing the ROC curves in the upper plot (A) of Figure 3 are all based on the k-means method. In the lower plot (B), circles and x-signs mark points defined by values of DEGs resulting from applying thresholds following from our adaptive Gaussian mixture algorithms (top3 corresponds to circles and k-means corresponds to the x sign).

In Table 3, we additionally present comparisons of different gene filtration methods, on the basis of two indexes describing the power of DEGs detection, the area under the ROC curve (AUC) and the sensitivity of DEGs search at the 10% FDR level.

**Table 3:** Comparison of power to detect DEGs between algorithms using different filtering methods applied to the spike-in dataset

**Method**	**NF**	**S_50**	**AS**	**V_50**	**AV**	**LV_50**	**ALV**	**PVAC**
AUC	12.60	12.57	12.73	12.68	13.11	12.96	11.94	**13.17**
Sensitivity	69.03	69.62	72.54	69.59	**73.37**	71.33	63.23	72.00

Data illustrated by two indexes: the area under the ROC curve in Figure 3 (AUC) from FDR equal 0 to 20% and sensitivity at 10% FDR.

**Figure 4:**
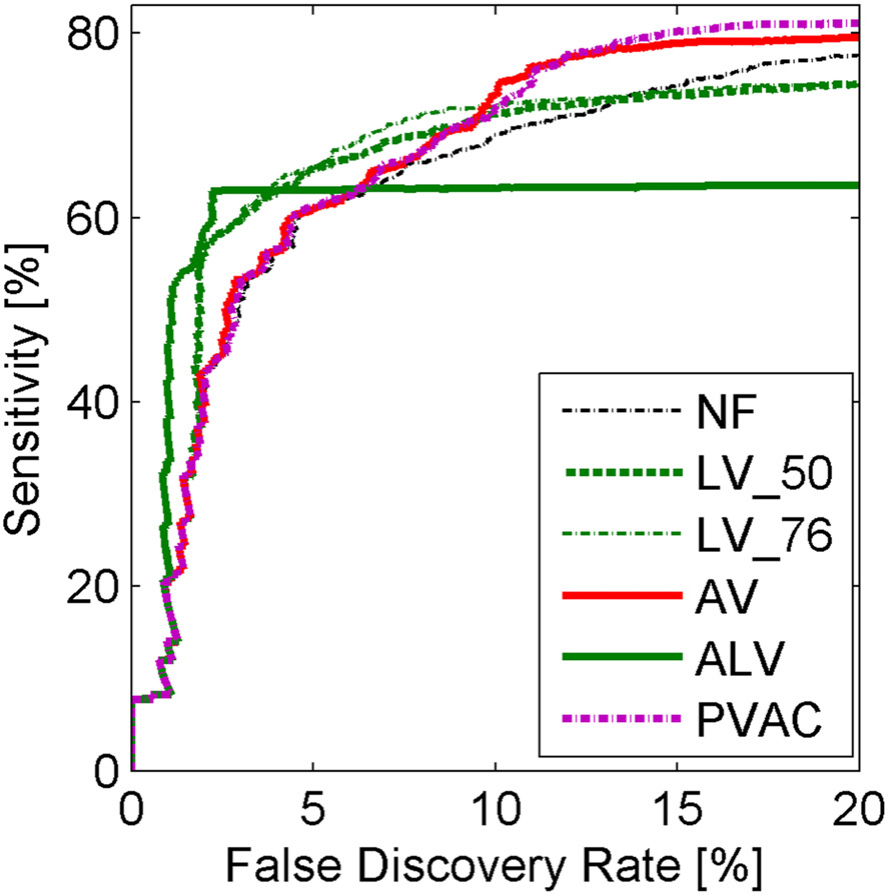
**Comparison of methods for discovery of DEGs based on ALV and AV filtering to PVAC filtering algorithm.** ROC curves for different filtering methods for spike-in dataset. Different colors correspond to different filtering types of filters: red color is assigned to the filtration in sample variance domain, blue – sample mean, green – sample variance in log scale, purple – PVAC method, and black – no filtration. Different line styles correspond to different methods: solid line shows the results for adaptive filtering, dashed and dashdot line – fixed threshold filtering. Percentage of the removed genes after PVAC filtering is 76.2.

In the second step of the analysis of the spike-in dataset we compare the results of applying ALV and AV filtering methods to the PVAC method proposed by Lu et al. [10]. In Figure 4 we present (in purple) a ROC curve corresponding to PVAC filtering method and, for comparison, by using different colors, ROC curves corresponding to ALV and AV filtering methods, and LV filtering methods with two different thresholds. Here ALV and AV filtering methods are based on k-means method. The application of the PVAC method results in the removal of genes except those which belong to the first principal component. In different datasets, the proportions between filtered out (removed) and retained genes are therefore different. In Figure 4 (along with other ROC curves) we also plot ROC curves corresponding to LV methods with percentages of removed genes equal to those obtained when using the PVAC method (LV_76).

**Figure 5:**
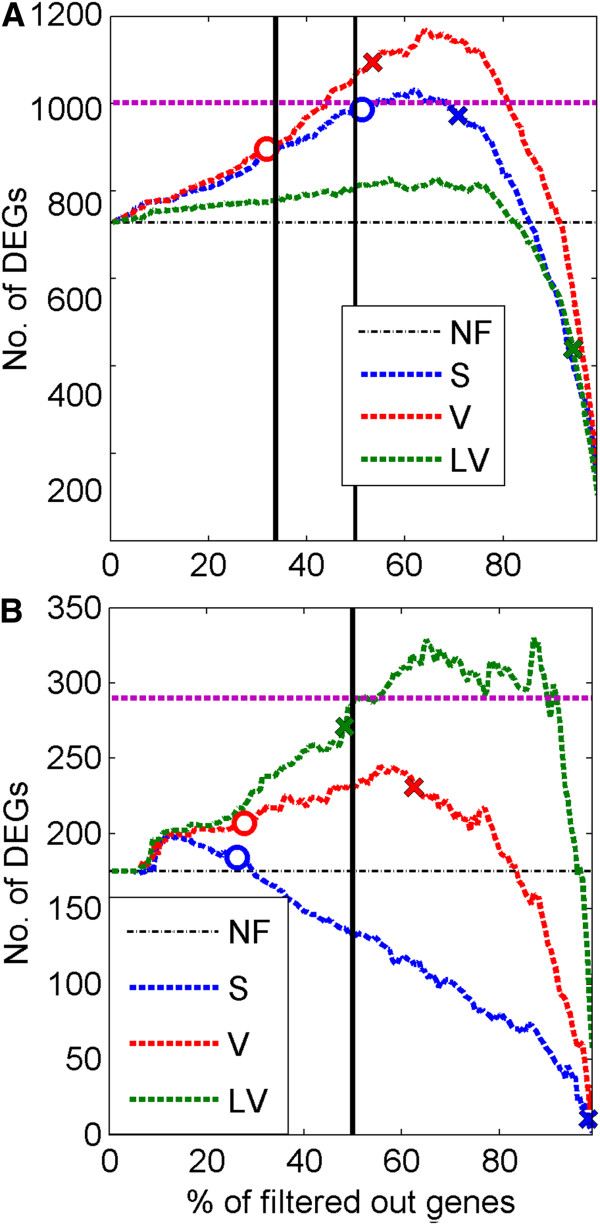
**Comparison of different filtering methods on diabetes and leukemia data.** Numbers of genes called DEGs (found by using *t*-test and q value correction for FDR) versus percentages of genes filtered out. Upper plot (**A**): diabetes dataset, lower plot (**B**): leukemia dataset. Different colors correspond to different filtering types of filters: red color is assigned to the filtration in sample variance domain, blue – sample mean, green – sample variance in log scale, and black – no filtration. Circles and x-signs on the plot correspond to points given by percentages following from our adaptive filtering methods (top3 and k-means respectively) and corresponding values of the DEGs. Top3 method for the ALV filter for the data sets in both plots **A** and **B** is equivalent to NF because we have only 2 Gaussian components in the mixture distribution. For the top3 method percentages of removed genes after AS and AV filtering are 51.3 and 31.9, respectively, in the rat diabetes dataset and 26.2 and 27.7, respectively, in the leukemia dataset. For the k-means method percentages of removed genes after AS, AV and ALV filtering are 70.1, 53.4 and 93.2, respectively, for the rat diabetes dataset and 98.2, 62.5 and 48.35, respectively, for the leukemia dataset. In the upper plot (**A**) the 34% threshold used in (6), 50% threshold and in the lower plot (**B**) 50% threshold used in (7) are marked with black vertical lines. Gene level related to the use of PVAC method is marked by purple horizontal dashed line. Estimated proportions of EEGs in the two datasets are as follows. Rat diabetes dataset: 0.968 (AS, top3 method), 0.971 (AV, top3 method), 0.968 (AS, k-means method), 0.964 (AV, k-means method), 0.985 (ALV, k-means method), 0.967 (PVAC). Leukemia dataset: 0.985 (AS, top3 method), 0.983 (AV, top3 method), 0.999 (AS, k-means method), 0.982 (AV, k-means method), 0.978 (ALV, k-means method), 0.977 (PVAC).

The assessment of the power of different methods to discover DEGs in the rat diabetes dataset and the leukemia dataset is based on the method proposed in [8] of counting numbers of null hypotheses, which can be rejected under a given constraint on FDR, assumed equal to 5%. In Figure 5 in the upper panel (A) we present the plots of the numbers of genes called DEGs versus percentages of genes filtered out for the diabetes dataset and in the lower panel (B) analogous plots for the leukemia dataset. Genes are called DEGs based on the *t*-test with q-value correction method for FDR. Three different curves correspond to filtrations based on sample means, variances and variances calculated on the expression values in log2 scale. Circles and x-signs mark points defined by the values of DEGs resulting from applying thresholds following from our adaptive Gaussian mixture algorithms (top3 corresponds to circles and k-means corresponds to the x sign). The black vertical line represents the threshold proposed in [7] on the basis of registration of “Absent” calls returned by the Affymetrix MAS 5.0 signal pre-processing procedure. If the PVAC filtering method is used in the first step of DEGs discovery then, in the second step, for diabetes data 1,002 null hypotheses can be rejected under a 5% constraint on FDR and for leukemia data the analogous number is 290. These numbers are marked by horizontal dashed lines.

## Discussion

### Comments to comparisons

For the artificially created dataset, we see from the plots in Figure 2 that in the analyzed range of FDR, all of the filtering methods increase median the sensitivity of DEGs search compared to the situation with no filtering. The increase of the proportion of EEGs (Figure 2, lower panel (B)) leads to the decrease of sensitivity of all methods. Based on the comparisons of plots in Figure 2 we conclude that AS is the best filter for the simulated dataset. One can notice that adaptive filtering by signal performs best in terms of sensitivity versus FDR because the method simulation of the expression signal values meets the assumptions of the S method more closely than filtering by variance, which is a limitation of the simulation methodology used here. Apart from using adaptive approach based of Gaussian mixture decompositions into fixed number of 2 Gaussian components (reported in Figure 2) we have also tested the second approach, where the number of Gaussian components was assumed unknown and was estimated by using BIC criterion and pools of removed and retained genes were determined by using either top3 or k-means methods. For each of 11 values of proportions of EEGs, which are distributed linearly in the range 70%-95%, we have performed 50 simulations (550 simulations in total). The simulation scenario was the same as that reported in Figure 2; the only difference was that instead of using a fixed number of components we assumed unknown number of components and estimate it using BIC criterion. The number of Gaussian components, different for each of 50 simulations and different for different proportions of EEGs and DEGs, varied from 2 to 4 (3 in 92% of cases) in the AS method, and from 4 to 5 (4 in 85% of cases) in the AV method. Consistently to the simulation scenario, in the decompositions of sample means there were always two dominating components (representing EEGs and DEGs). Results of applying different filtration methods to simulated data for the case when the proportion between informative and non-informative genes was set to 85% EEGs versus 15% DEGs are presented in Table 2. Again, the AS filtration method was outperforming other filtration methods. When we used k-means method for AS filter ROC curves and plots of sensitivity were very similar to those presented in Figure 2. However, due to the small number of mixture components the use of top3 method for simulated data rarely increased sensitivity of finding DEGs. In the majority of cases AS method was used on 3 components model, so introducing top3 method gave the same results as NF. The mixture decompositions of the distributions of sample variances were most often built of 4 components and retaining 3 components gave results similar to V_50. AV filter gave similar results to V_50 filter for the range of values of proportion of EEGs 70% - 80%. When we further increased the number of EEGs we filter out too many genes with AV, which resulted in decreasing sensitivity of finding DEGs. At EEGs = 90% the median sensitivity resulting from using V_50, was equal to 60.72% compared to median sensitivity 42.01% resulting from using AV.

For the spike-in dataset, where we use the F1 measure and ROC curves to compare filtering methods and show results in Figure 3, we observe that at low values of FDR the highest sensitivity is achieved by our ALV method (Figure 3, upper plot (A)). However, at higher values of FDR we see a flattening of the ROC curve for the ALV method. This shape of the FDR curve is related to the fact that the application of the ALV method leads to filtering out quite a high percentage (about 93%) of genes in this dataset. From Figure 3 we can notice that the use of the ALV method gives the worst sensitivity of finding DEGs. From plot (B) we notice that the methods k-means and top3 lead to the same result in AS and ALV filtration. From the plots of F1 indexes versus percentages of genes filtered out (the lower plot (B) of Figure 3) one can see that the threshold values obtained by using AV, AS and ALV methods are close to optimal i.e. close to values of filtering thresholds corresponding to maxima of the F1 measure. In comparisons of adaptive to fixed threshold methods (AS to S_50, AV to V_50 and ALV to LV_50) we conclude that AS outperforms S_50, AV outperforms V_50 but ALV led to worse result than LV_50. Also both AV and AS outperform the no filtering method. As in [10] we also check influence of filtering, with a smaller number of replicates (data not shown). In all cases adaptive filtering increases the sensitivity of finding DEGs. The general conclusion is that when the number of replicates is smaller, the increase is higher. In the spike-in dataset analysis filters based on variances give better results than those based on means. Comparisons of the PVAC method to variance based filters, shown in Figure 4 leads to the conclusion that PVAC is indeed a highly effective method, but still similar to AV. In the range of low values of FDR, PVAC is outperformed by our ALV method.

Contemplation of ROC curves in Figures 3 (upper plot) and 4 leads to an observation that when FDR changes (increases), relations between sensitivities of different methods can become inverted. If the increase of FDR was continued to very high values (exceeding the ranges in Figures 3 and 4), then the highest sensitivity would be achieved by no filtering (NF) method. This shows that all filtering methods (except NF) are under risk of committing type II statistical errors of removing some proportion of true DEGs and that different methods can offer different compromises between sensitivities and FDR. When indexes like F1 or AUC are used, some methods can fully outperform others, as discussed above.

The plot in the upper panel (A) of Figure 5 demonstrates that for the rat diabetes dataset, filtering thresholds found by using our adaptive methods are (again) close to optimal with respect to the measure given by the number of genes that can be called DEGs. S and V gene filtering methods based on adaptive thresholds are superior to the method of using 34% threshold level resulting from “Absent” calls of probe sets, analyzed in [7]. The use of the adaptive version of the LV method leads to poor results. We can explain this by contemplating the histogram shown in Figure 1H, which does not exhibit distinctive Gaussian components. In this situation, there is a high overlap between two components detected, which leads to the removal of excessive number of genes called uninformative. Concerning the comparison of our adaptive methods to the PVAC method, the level of 1,002 genes obtained by using the PVAC method was outperformed by our AV method.

The comparison between upper and lower plot in Figure 5 shows that the choice of the type of filter can be crucial for the obtained results. For the leukemia dataset the best result is obtained after using the ALV method, which is the worst choice for the rat diabetes dataset. This stems from the fact that in the diabetes dataset the DEGs belong to the group of highly expressed genes (across all treatments), which is not true for the leukemia dataset. Strictly, for no filtering case in diabetes dataset, median of mean expression of estimated DEGs across all treatments is equal to 9.06 and of estimated EEGs is equal to 5.92 (1.53 times smaller than for DEGs). Median of variance of expression on the log2 scale of estimated DEGs across all treatments is equal to 0.119 and of estimated EEGs is equal to 0.061 (1.95 times smaller than for DEGs). For no filtering case in leukemia dataset, median of mean expression of estimated DEGs across all treatments is equal to 5.99 and of estimated EEGs is equal to 5.49 (1.09 times smaller than for DEGs). Median of variance of expression on the log2 scale of estimated DEGs across all treatments is equal to 0.628 and of estimated EEGs is equal to 0.102 (6.15 times smaller than for DEGs).

### Assessment of the proposed methodology

Our adaptive filtering methods based on Gaussian mixture decompositions do not use sample class labels. Combined with the *t*-test they satisfy the “marginal independence” condition [8] mentioned in the Introduction section, since they only use sample means or variances corresponding to gene expressions signals. Therefore we consider the proposed methodology as a reliable approach for gene filtering.

The representation of the probability distribution function as a mixture of components can be related to certain hypotheses concerning measured signals. Components of signals defined by means or variances can be interpreted as corresponding to technical (measurement) noise, biological variation or to biological or cellular processes. Decomposition of the distribution of the signal x into a mixture of (Gaussian) components is based on well-developed methods of statistical modeling [16]. Different variants of methods of decompositions of signal distribution into mixtures of components were already successfully applied to several problems of analyses of DNA microarray data, examples of related papers are [19-24]. In [20-23] mixture decompositions are used for unsupervised clustering of microarray data. Authors of paper [19] propose mixture models for assessing differential expression between samples in microarray data. Lee et al. [24] use a mixture model for analysis of replicated microarray experiments. In this paper, we extend the range of applications of the mixture decomposition methodology to the problem of filtering genes in DNA microarrays.

The mechanism of adaptation related to the mixture decomposition approach can be intuitively explained as follows. If in the analyzed data there are many genes or probe sets, highly corrupted by noise, with low levels of signal to noise ratios, then there would most probably exist corresponding Gaussian components with low values of x signal and quite high component weights. These components will be removed by our filtration procedure. Both of the two proposed selection methods, top3 and k-means, have adaptation potential. It seems, however, that the k-means method can lead to better results as seen in Figures 3 and 5.

## Conclusions

The power of our adaptive method for improving detection of DEGs is compared to the results reported in earlier papers [7,10]. Efficiencies of methods for improving DEGs detection power in microarray data are compared by using two datasets, in which the status of each gene is known. Adaptive filtering repeatedly takes the highest places in comparisons of detection powers by different indexes (ROC curves, the F1 index, and the AUC index). The efficiencies of different two-step methods for improving DEGs detection power are also estimated and compared for the rat diabetes and leukemia datasets, where the status of genes is not known, by comparing the numbers of the discovered DEGs for the same limits on FDR. The numbers of DEGs found by using adaptive filtering (AV and ALV respectively) belong to the highest among the compared methods. In conclusion, the number of genes to filter out by overall mean and variance should not be fixed, but rather found based on probe set signal properties (distributions), and the methodology for setting adaptive thresholds based on mixture decompositions is competitive compared to other gene filtering approaches.

## Competing interests

The authors declare that they have no competing interests.

## Authors’ contributions

MM - performed all computational work related to samples preprocessing, filtering and classification and contributed with several hypotheses concerning possible methodologies for formulating the conditions for removing and retaining components. He also helped in writing the manuscript. RJ – elaborated and implemented procedures for data normalization and storage. AP – helped in writing the manuscript and formulating conclusions. JP – outlined the research and helped in writing and organizing the manuscript. All authors read and approved the final manuscript.
